# Comparative efficacy of total knee arthroplasty versus unicompartmental knee arthroplasty in obese patients with medial knee osteoarthritis

**DOI:** 10.3389/fmed.2026.1782061

**Published:** 2026-04-09

**Authors:** Mingyue Fan, Jinxi An, Yangping Tang, Binbin Gu

**Affiliations:** 1 Fuyang Tumor Hospital, Fuyang, Anhui, China; 2 The First Affiliated Hospital of Anhui University of Science and Technology (Huainan First People’s Hospital), Huainan, Anhui, China

**Keywords:** kneefunction, lower limb alignment, obesity, total knee arthroplasty, unicompartmental knee arthroplasty

## Abstract

**Background:**

This study aimed to compare the efficacy of total knee arthroplasty (TKA) and unicompartmental knee arthroplasty (UKA) in the treatment of medial knee osteoarthritis in obese patients.

**Methods:**

A total of 75 patients who underwent knee arthroplasty at our institution between June 2022 and June 2024 were included. Based on physician-patient communication, 34 patients were treated with UKA and 41 with TKA. General patient data, surgical-related indicators, and radiographic and follow-up data were compared between the two groups.

**Results:**

The UKA group demonstrated superior outcomes in terms of intraoperative blood loss, incision length, and postoperative drainage volume compared to the TKA group (*P* < 0.001). No statistically significant differences were observed in VAS scores or operative time between the two groups (*P* > 0.05). The UKA group showed significantly better HSS and WOMAC scores than the TKA group (*P* < 0.001). At the final follow-up, both groups exhibited significant improvements in the medial proximal tibial angle, posterior tibial slope, and femorotibial angle compared to preoperative values (*P* < 0.05).

**Conclusion:**

UKA and TKA demonstrated comparable efficacy in restoring lower limb alignment and improving knee function. However, UKA offered distinct advantages in reducing intraoperative blood loss, incision length, and postoperative drainage. With strict adherence to surgical indications, UKA may be prioritized to enhance patient satisfaction following knee arthroplasty.

## Background

Knee osteoarthritis (KOA) is a common degenerative joint disease and represents one of the leading causes of pain, functional impairment, and disability among middle-aged and elderly populations worldwide (1). With the accelerating trend of global population aging and the continuous rise in obesity rates, the prevalence of KOA has shown a marked increase, imposing a growing burden on public health systems. Among the various subtypes of KOA, medial compartment knee osteoarthritis is particularly prevalent. Obesity, as an independent risk factor for KOA, has been widely substantiated in its association with the onset and progression of the disease (2). For end-stage medial compartment KOA refractory to conservative treatment, the primary surgical interventions currently include total knee arthroplasty (TKA) and unicompartmental knee arthroplasty (UKA) (3). However, the impact of obesity on postoperative outcomes following UKA remains a subject of ongoing debate in the field of orthopedics (4). This study systematically compares the therapeutic efficacy of TKA and UKA in obese patients, aiming to provide reliable evidence-based medical data to assist clinicians in selecting the most appropriate surgical approach for this patient population.

## Materials and methods

This retrospective study, conducted over a 2-year period from June 2022 to June 2024, evaluated 75 patients who underwent knee arthroplasty. Based on the surgical procedure performed, the patients were divided into two groups: the UKA group, consisting of 34 patients who received unicompartmental knee arthroplasty, and the TKA group, comprising 41 patients who underwent total knee arthroplasty.

### Case selection

Inclusion criteria were:

Diagnosed with medial compartment knee osteoarthritis;

Knee flexion contracture ≤ 15°, varus deformity ≤ 10°, and range of motion ≥ 90°;

Intact ligamentous function of the knee;

Body mass index (BMI) ≥ 28 kg/m^2^ (5);

Complete follow-up data available for a minimum of 12 months postoperatively.

Exclusion Criteria were:

Multi-compartment osteoarthritis;

Knee flexion contracture > 15°, varus deformity > 10°, or range of motion < 90°;

Concomitant joint pathologies such as rheumatoid arthritis, gout, tumors, or infections;

Previous history of knee surgery;

Severe osteoporosis;

Unwillingness to participate in the follow-up process.

### Surgical technique

Unicompartmental Knee Arthroplasty (UKA): All UKA procedures were performed by the same senior arthroplasty surgeon. Following the induction of satisfactory anesthesia, the patient was positioned supine. The affected limb was routinely prepared and draped. A standard medial parapatellar approach was utilized to access the knee joint. The joint cavity was entered, and partial resection of the infrapatellar fat pad was performed to adequately expose the anteromedial tibial plateau. Intraoperative inspection confirmed wear of the articular cartilage on the anteromedial tibia and distal femur, with the presence of osteophytes in the intercondylar notch. The anterior cruciate ligament was noted to be functionally intact, with no evidence of cartilage wear in the lateral compartment and integrity of the collateral ligaments. Synovial tissue and osteophytes were debrided. A tibial cutting guide was positioned anteriorly on the tibia, abutting the insertion of the anterior cruciate ligament, and set to maintain a posterior tibial slope of 5–6°. The appropriate implant size was selected based on the planned resection. Subsequent femoral preparation involved milling of the distal femur. The medial meniscus was resected, and the flexion and extension gaps were measured. Bone resections were performed accordingly. A tibial template was positioned to guide the precise bone cut and keel preparation. Trial components (tibial, femoral, and meniscal bearing) were then inserted. Knee stability and bearing impingement were assessed, and gap balance was confirmed throughout the range of motion. The definitive tibial and femoral components were cemented in place, excess cement was removed, and final stability was verified. The wound was irrigated, a single intra-articular drain was placed, and closure was performed in layers followed by application of a compressive dressing.

Total Knee Arthroplasty (TKA): After the induction of anesthesia, the patient was positioned supine with the surgical limb prepared and draped. A standard midline skin incision was made, followed by a medial parapatellar arthrotomy. Joint exploration revealed patellofemoral cartilage wear, peripheral osteophytosis, loss of articular cartilage on weight-bearing surfaces, and femoral condylar osteophytes. Osteophytes, hypertrophic synovium, and both the anterior and posterior cruciate ligaments, along with the medial and lateral menisci, were resected. The tibia was anteriorly subluxated, and the proximal tibial cut was performed. The distal femoral cut was made with 5° of valgus alignment. Trial components were inserted to assess range of motion, implant sizing, and soft-tissue balance. After satisfactory trialing, bone cement was applied, and the definitive tibial and femoral components were sequentially implanted under pressure. The polyethylene insert was inserted after cement hardening. Final assessment confirmed satisfactory joint mobility, limb alignment, and patellar tracking. The surgical site was irrigated, a single drain was placed, and a layered closure with a compressive dressing concluded the procedure.

### Postoperative management

Standard prophylactic antibiotic therapy was administered postoperatively. During hospitalization, subcutaneous injections of low-molecular-weight heparin calcium were administered for deep vein thrombosis prophylaxis, with subsequent transition to oral rivaroxaban upon discharge. Following the resolution of anesthesia, patients were instructed to perform isometric quadriceps contractions and ankle plantarflexion-dorsiflexion exercises to enhance lower limb circulation and reduce edema. On the second postoperative day, patients were assisted in ambulation and commenced passive knee flexion exercises using a continuous passive motion machine, administered twice daily.

### Outcome measures

#### Perioperative parameters

Surgical duration, incision length, intraoperative blood loss, and postoperative drainage volume were systematically recorded for all patients.

#### Joint function assessment

Functional outcomes were evaluated using established scoring systems and physical examinations: the Visual Analog Scale (VAS) for pain (6), the Hospital for Special Surgery (HSS) Knee Score (7), active knee range of motion (ROM) in flexion-extension (8), and the Western Ontario and McMaster Universities Osteoarthritis Index (WOMAC) (9). Complications occurring during the follow-up period, including surgical site infection, joint dislocation, and the necessity for revision surgery, were meticulously documented.

#### Radiographic evaluation of lower limb alignment

Standardized radiographic measurements were performed to assess coronal and sagittal plane alignment. The Femorotibial Angle (FTA) was defined as the angle formed between the anatomical axes of the femur and the tibia in the coronal plane. The Posterior Tibial Slope (PTS) was measured on a true lateral radiograph of the knee in full extension as the angle between the medial tibial plateau and the proximal tibial anatomical axis. The Medial Proximal Tibial Angle (MPTA) was defined as the medial angle between the tibial anatomical axis and the knee joint line in the coronal plane.

### Statistical analysis

All statistical analyses were performed using SPSS version 26.0 (IBM Corp., Armonk, NY, United States). Continuous variables are presented as mean ± standard deviation (Mean ± SD) if they followed a normal distribution; otherwise, they are expressed as median with interquartile range [M (Q1, Q3 )]. Categorical data are summarized as frequency and percentage [n (%)].For group comparisons, independent samples *t*-tests were applied for normally distributed continuous variables, while the Mann-Whitney U test was used for non-normally distributed continuous variables. Comparisons of categorical variables between groups were conducted using the chi-square (χ^2^) test. A two-tailed *p*< 0.05 was considered statistically significant for all tests.

## Results

### Baseline characteristics

A retrospective analysis was conducted on 75 eligible patients enrolled between June 2022 and June 2024, all of whom possessed complete clinical and follow-up data. As summarized in Table 1, no statistically significant differences were observed between the UKA group and the TKA group in terms of age, body mass index, sex distribution or affected side. These findings confirm that the baseline characteristics were well-balanced and comparable between the two groups. The study protocol received approval from the Institutional Ethics Committee of our hospital.

**TABLE 1:** Comparison of baseline characteristics between UKA and TKA groups.

Variable	UKA group (*n* = 34)	TKA group (*n* = 41)	Statistic	*P*-value
Age (years), M (Q1, Q3)	63.00 (61.25, 65.00)	63.00 (61.00, 65.00)	*Z* = −0.05	0.957
BMI (kg/m^2^), M (Q1, Q3)	32.50 (31.50, 33.55)	33.40 (31.50, 33.60)	*Z* = −1.47	0.143
Sex, n (%)			χ^2^ = 0.07	0.788
Male	9 (26.47)	12 (29.27)		
Female	25 (73.53)	29 (70.73)		
Affected side, n (%)			χ^2^ = 3.42	0.064
Right	11 (32.35)	22 (53.66)		
Left	23 (67.65)	19 (46.34)		

### Perioperative data

As detailed in Table 2, no statistically significant difference was observed in operative time between the two groups (*P* > 0.05). Conversely, the UKA group demonstrated significant superiority over the TKA group in terms of intraoperative blood loss, incision length, and postoperative drainage volume (*P* < 0.001).

**TABLE 2:** Comparison of perioperative outcomes between UKA and TKA groups.

Variable	UKA group (*n* = 34)	TKA group (*n* = 41)	Statistic	*P*-value
Operative time (min), Mean ± SD	80.41 ± 5.30	81.54 ± 4.46	*t* = −1.00	0.321
Intraoperative blood loss (mL), Mean ± SD	110.24 ± 5.41	135.90 ± 12.17	*t* = −12.14	**< 0.001**
Incision length (cm), M (Q1, Q3)	6.55 (6.40, 6.80)	8.40 (8.30, 8.60)	*Z* = −7.41	**< 0.001**
Postoperative drainage volume (mL), M (Q1, Q3)	167.00 (160.00, 180.00)	185.00 (180.00, 186.00)	*Z* = −5.05	**< 0.001**

Bold values represent statistically significant differences (*P* < 0.05).

### Clinical outcomes

As detailed in Table 3, no statistically significant differences were observed in the VAS scores and knee flexion range of motion between the two groups either preoperatively or at the final follow-up (all *P* > 0.05). Preoperative HSS scores were comparable between the groups (*P* > 0.05). At the final follow-up, both groups exhibited a significant increase in HSS scores compared to their preoperative levels. Notably, the UKA group demonstrated significantly higher HSS scores than the TKA group at this terminal assessment (*P* < 0.05). Similarly, while no significant intergroup difference was found in preoperative WOMAC scores (*P* > 0.05), both groups showed significant improvement with lower scores at the final follow-up. The UKA group achieved superior outcomes, registering significantly lower WOMAC scores compared to the TKA group (*P* < 0.05).

**TABLE 3:** Comparison of functional outcomes between UKA and TKA groups.

Outcome measure	Timepoint	UKA group (*n* = 34)	TKA group (*n* = 41)	*Z*-value	*P*-value
HSS score	Preoperative	55.00 (54.00, 60.50)	55.00 (53.00, 58.00)	−0.80	0.427
	Postoperative	79.00 (76.00, 81.00)	74.00 (71.00, 76.00)	−5.44	**< 0.001**
*Z*-value		−5.089	−5.582		
*P*-value		**< 0.001**	**<0.001**		
Knee flexion ROM (°)	Preoperative	105.00 (102.00, 108.00)	105.00 (102.00, 107.00)	−0.39	0.700
	Postoperative	130.00 (120.00, 135.00)	130.00 (125.00, 135.00)	−1.12	0.263
*Z*-value		−5.089	−5.581		
*P*-value		**< 0.001**	**<0.001**		
WOMAC score	Preoperative	72.00 (69.00, 74.75)	74.00 (72.00, 75.00)	−1.78	0.075
	Postoperative	24.00 (23.00, 28.00)	29.00 (28.00, 32.00)	−4.81	**< 0.001**
*Z*-value		−5.089	−5.586		
*P*-value		**< 0.001**	**<0.001**		
VAS score	Preoperative	6.00 (5.00, 6.00)	6.00 (5.00, 6.00)	−0.21	0.832
	Postoperative	2.00 (1.25, 2.00)	2.00 (2.00, 3.00)	−0.82	0.410
*Z*-value		−5.134	−5.637		
*P*-value		**< 0.001**	**<0.001**		

Bold values represent statistically significant differences (*P* < 0.05).

### Radiological results

As detailed in Table 4, no statistically significant differences were observed in the medial proximal tibial angle (MPTA) or the femorotibial angle (FTA) between the two groups at either the preoperative assessment or the final postoperative follow-up (all *P* > 0.05). In contrast, the posterior tibial slope (PTS) was significantly smaller in the UKA group compared to the TKA group at both timepoints (all *P* < 0.05).

**TABLE 4:** Comparison of radiographic outcomes between UKA and TKA groups.

Radiographic parameter	Timepoint	UKA group (*n* = 34)	TKA group (*n* = 41)	Statistic	*P*-value
MPTA (°)	Preoperative	76.00 (74.25, 77.00)	76.00 (76.00, 79.00)	*Z* = −1.85	0.064
	Postoperative	91.00 (90.00, 93.00)	92.00 (90.00, 94.00)	*Z* = −1.40	0.162
*Z*-value		−5.093	−5.586		
*P*-value		**< 0.001**	**<0.001**		
PTS (°)	Preoperative	8.50 (8.40, 8.60)	8.60 (8.50, 8.70)	*Z* = −2.23	**0.026**
	Postoperative	7.80 (7.70, 7.90)	7.90 (7.80, 8.10)	*Z* = −2.31	**0.021**
*Z*-value		−5.111	−5.607		
*P*-value		**< 0.001**	**<0.001**		
FTA (°)	Preoperative	182.00 (179.25, 184.00)	183.00 (182.00, 186.00)	*Z* = −1.71	0.088
	Postoperative	171.03 ± 1.93	170.15 ± 2.52	*t* = 1.68	0.098
*Z*-value		−5.110	−5.584		
*P*-value		**< 0.001**	**<0.001**		

Bold values represent statistically significant differences (*P* < 0.05).

### Complications

**FIGURE 1:**
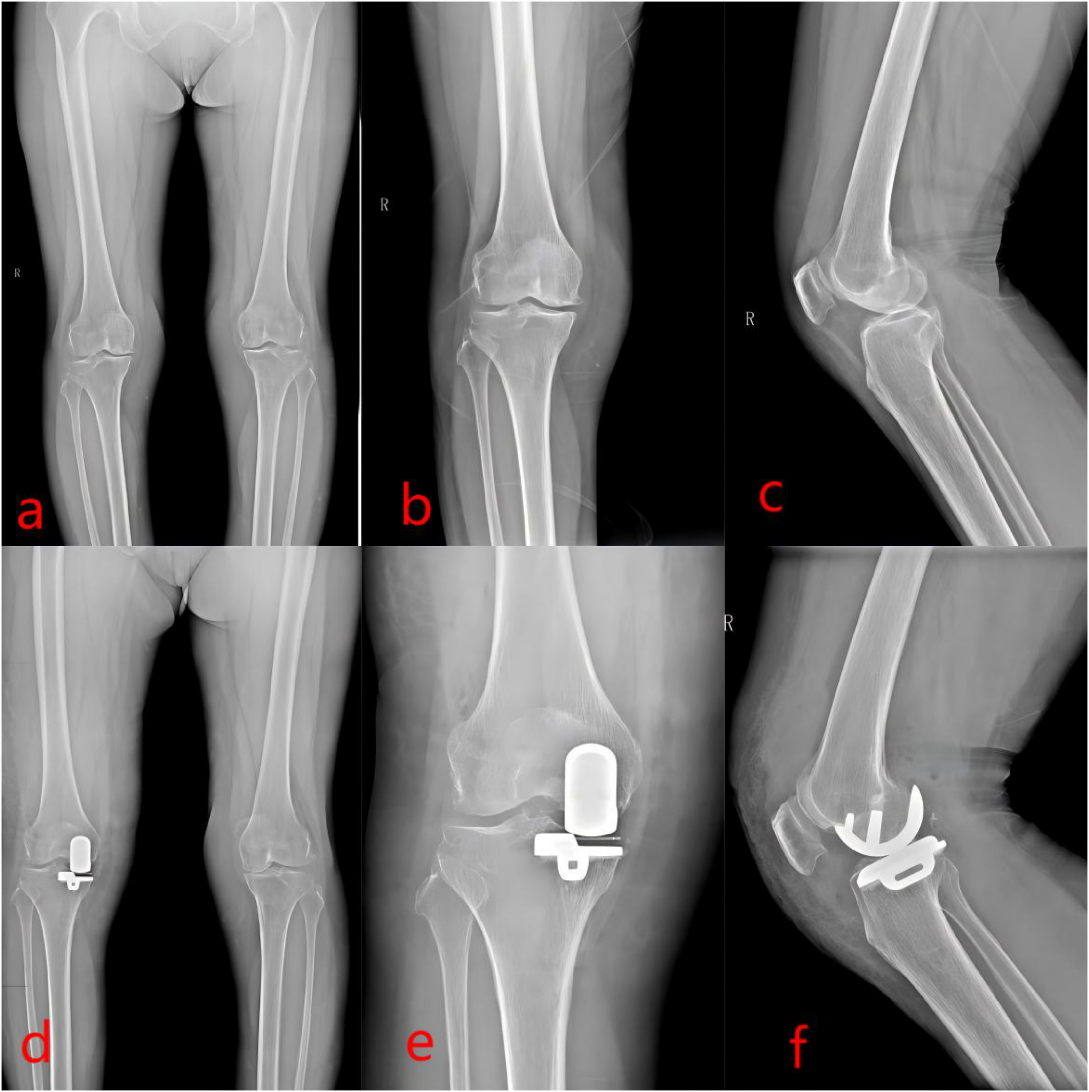
Unicompartmental knee arthroplasty (UKA). This figure shows a 65-year-old female patient. **(a–c)** Preoperative anteroposterior and lateral X-rays of the right knee joint and full-length X-rays of the lower extremity demonstrate the manifestations of knee osteoarthritis (KOA) in the right knee joint, with narrowing of the medial compartment and mild varus deformity of the knee joint. **(d–f)** Postoperative anteroposterior and lateral X-rays of the right knee joint and full-length X-rays of the lower extremity show that the prosthesis is well fixed without any sign of loosening. The varus deformity and narrowing of the knee joint have been corrected, and the mechanical axis of the lower extremity has been significantly improved compared with that before the operation.

**FIGURE 2:**
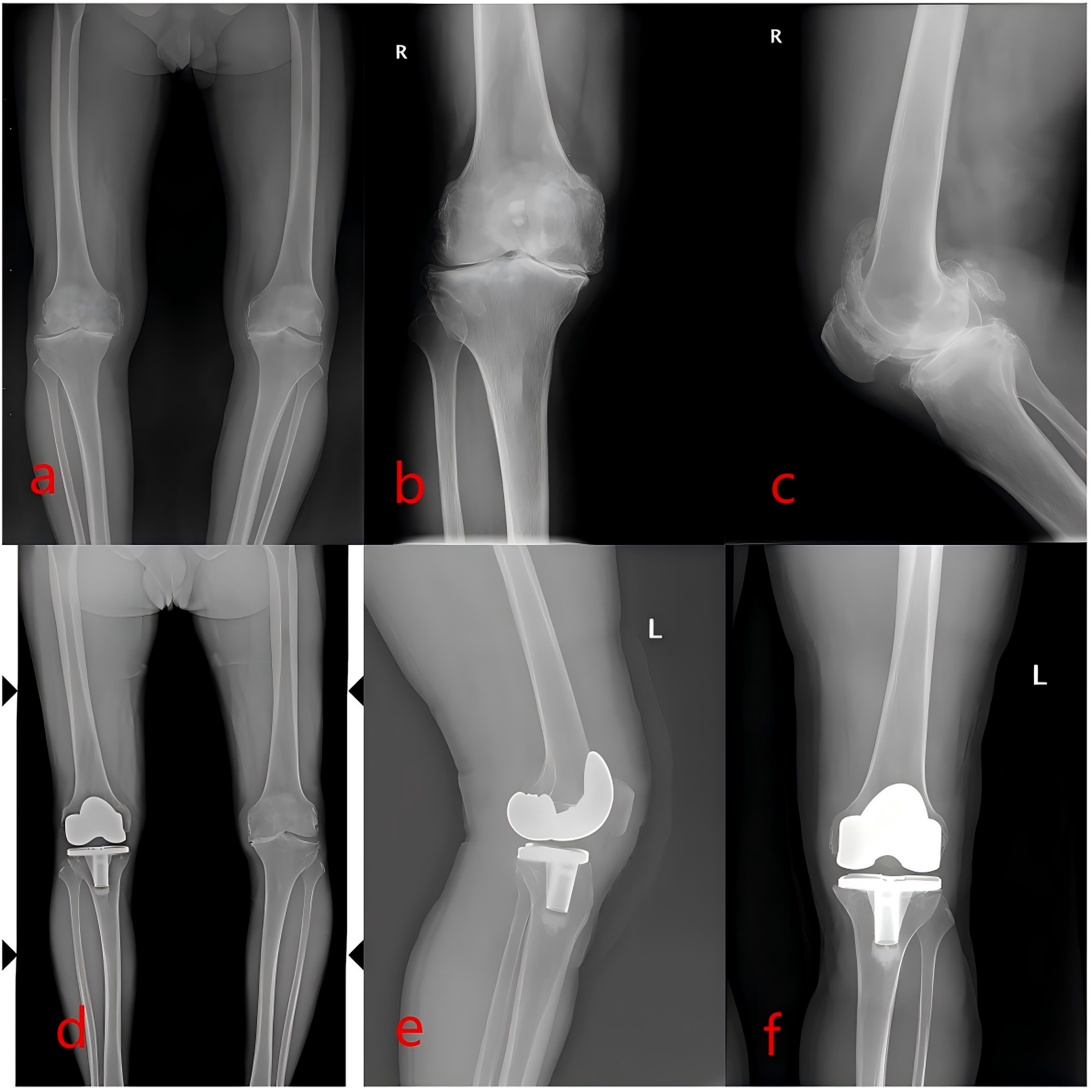
Total knee arthroplasty (TKA). Male patient, 62 years old. **(a–c)** Preoperative anteroposterior and lateral views of the right knee joint and full-length X-ray of the lower extremity showed the KOA manifestations of the right knee joint, with severe narrowing of the medial compartment of the knee joint and genu varum. **(d–f)** Postoperative anteroposterior and lateral views of the right knee joint and full-length X-ray showed that the prosthesis was well fixed without any loosening signs. The genu varum and narrowing of the knee joint were corrected, and the lower extremity alignment was improved compared to that before the operation.

All surgical incisions healed primarily, with no instances of surgical site infection or deep vein thrombosis reported in either group. Postoperative radiographic evaluation revealed improved knee alignment in both cohorts compared to preoperative status, with significant correction of varus deformity (Figure 1). At the final follow-up, none of the patients exhibited evidence of prosthesis loosening or dislocation. Furthermore, no progressive degeneration was observed in the lateral compartment of the UKA group compared to preoperative findings (Figure 2).

## Discussion

For the treatment of end-stage knee osteoarthritis, total knee arthroplasty (TKA) and unicompartmental knee arthroplasty (UKA) represent two important surgical options. Compared with TKA, UKA offers the advantages of being minimally invasive, preserving normal bone structures and ligaments to the greatest extent, and facilitating faster postoperative recovery (10, 11). Early perspectives considered obesity a contraindication for unicompartmental knee arthroplasty (UKA), primarily due to concerns regarding increased risks of prosthesis loosening and revision, as well as lower implant survival rates in obese patients (12). However, with continuous improvements in prosthesis design, increased surgical proficiency, and refined indications, UKA has been increasingly utilized in patients with knee osteoarthritis (KOA) (13–15). Nevertheless, the impact of obesity on postoperative outcomes following UKA remains a subject of ongoing debate in orthopedics. Xu et al. (16) conducted a follow-up study of at least 10 years and reported poorer clinical outcomes and significantly higher revision rates among obese patients. In contrast, Plate et al. (17), in a study of 212 UKA patients with a mean follow-up of 12 years, found that obesity did not affect the long-term survival or clinical outcomes of mobile-bearing UKA. Similarly, a meta-analysis by Lua et al. (18) comparing obese and non-obese patients after UKA revealed no statistically significant differences in overall complications, infections, deep vein thrombosis, embolism, prosthesis loosening, or revision rates.

In the present study, all incisions healed primarily, with no occurrences of infection, deep vein thrombosis, prosthesis loosening, or revision in either group. This favorable outcome may be attributed to the preservation of native knee structures in UKA, which potentially reduces complication risks. Although TKA involves more extensive bone and soft-tissue resection, standardized surgical protocols and established complication prevention systems—such as standardized osteotomy techniques and perioperative management—may mitigate the disadvantages associated with its more invasive nature. Additionally, patient-specific factors, postoperative care, and rehabilitation regimens also play critical roles in determining complication rates.

Our findings indicated that the UKA group exhibited significant advantages over the TKA group in terms of intraoperative blood loss, incision length, and postoperative drainage volume, consistent with the results reported by Huang et al. (19). This can be explained by the tissue-sparing nature of UKA, which involves replacement of only the affected compartment while preserving the anterior cruciate ligament and much of the native knee anatomy.

At the final follow-up, both groups demonstrated significant improvements in VAS, HSS, knee flexion range of motion, and WOMAC scores compared to preoperative values, indicating that both TKA and UKA effectively alleviated pain and enhanced functional recovery. These findings align with those reported by Liu et al. (20). The present study also revealed that the UKA group achieved superior HSS and WOMAC scores postoperatively compared to the TKA group. This finding can be attributed to the fact that UKA involves replacement of only the affected compartment without compromising the entire knee joint structure. Such a surgical approach helps preserve normal knee anatomy and function—including the joint capsule, ligaments, and muscles—thereby facilitating early postoperative functional exercises and contributing to better knee function outcomes. These results are consistent with the findings reported by Arirachakaran et al. (21) Conversely, Tille et al. (22) found no significant differences in HSS scores or knee range of motion between UKA and TKA patients at 2 years postoperatively, suggesting that the long-term clinical outcomes of UKA and TKA are comparable.

The precise restoration of lower limb alignment is a fundamental objective in knee arthroplasty, as it is directly correlated with long-term implant survival and clinical outcomes (23, 24). Whether achieved through the comprehensive realignment afforded by total knee arthroplasty (TKA) or the more limited correction enabled by unicompartmental knee arthroplasty (UKA), both techniques can effectively reestablish physiological lower limb alignment (23, 24). In the present study, no revision cases were reported during the follow-up period. Radiographic assessment at final follow-up revealed significant improvements in the medial proximal tibial angle (MPTA), posterior tibial slope (PTS), and femorotibial angle (FTA) compared to preoperative measurements in both groups, with no statistically significant differences observed between the UKA and TKA cohorts. These findings align with previous studies (25–27), indicating that both surgical approaches can effectively restore coronal plane alignment in patients with medial compartment knee osteoarthritis.

## Conclusion

In summary, both UKA and TKA yield satisfactory clinical outcomes in obese patients with medial compartment knee osteoarthritis. However, compared to TKA, UKA demonstrates superior outcomes in terms of intraoperative blood loss, incision length, postoperative drainage volume, and functional scores (HSS and WOMAC). Both procedures effectively improve lower limb alignment and restore knee range of motion.

## Limitations

Several limitations of this study should be acknowledged. The analysis did not fully account for potential confounding factors such as gender and prosthesis type. Although no failure cases were recorded, the relatively short follow-up period precludes definitive conclusions regarding the long-term impact of obesity on knee arthroplasty outcomes. Furthermore, the modest sample size warrants validation through future studies with larger cohorts and extended follow-up durations.

## Data Availability

The original contributions presented in this study are included in this article/supplementary material, further inquiries can be directed to the corresponding author.

## References

[1] Chen L Zhou H Gong Y Tang Y Su H Jin Z et al. How do muscle function and quality affect the progression of KOA? A narrative review. *Orthop Surg.* (2024) 16:802–10. 10.1111/os.14022 38438160 PMC10984828

[2] Zheng C Lu W Li Z Zhou J Chen D Wu Y. [Effect of body mass index on short- and medium-term effectiveness of unicompartmental knee arthroplasty]. *Zhongguo Xiu Fu Chong Jian Wai Ke Za Zhi.* (2020) 34:442–6. 10.21203/rs.3.rs-2141152/v1 32291978 PMC8171515

[3] Smith WB II Steinberg J Scholtes S Mcnamara IR. Medial compartment knee osteoarthritis: age-stratified cost-effectiveness of total knee arthroplasty, unicompartmental knee arthroplasty, and high tibial osteotomy. *Knee Surg Sports Traumatol Arthrosc.* (2017) 25:924–33. 10.1007/s00167-015-3821-3 26520646

[4] Campi S Papalia GF Esposito C Albo E Cannata F Zampogna B et al. Unicompartmental knee replacement in obese patients: a systematic review and meta-analysis. *J Clin Med.* (2021) 10:3594. 10.3390/jcm10163594 34441889 PMC8397050

[5] Li MY Luo YY Zhang P Chen W Zhang YQ Fang ZZ et al. [Interpretation of national clinical practice guideline on obesity management (2024 edition)]. *Zhonghua Yi Xue Za Zhi.* (2025) 105:1387–91. 10.1088/0256-307x/13/5/016 40340216

[6] Rizvi SB Ullah N Tahir M Ali Y Tahir S Alotaibi N et al. Robot-assisted total vs. unicompartmental knee arthroplasty: a systematic review and meta-analysis. *Turk J Surg.* (2025) 41:428–36. 10.47717/turkjsurg.2025.2025-5-14 41059591 PMC12687397

[7] Abdel Khalik H Nadeem SM Cruickshank M Chalmers BP Lanting B Wood TJ. Robotic-assisted total hip arthroplasty using the direct anterior approach: a systematic review and meta-analysis. *J Orthop.* (2025) 71:136–44. 10.1016/j.jor.2025.10.020 41246168 PMC12616034

[8] Waseem MH Abideen ZU Khan MH Tahir MF Mukhlis M Kakakhail A et al. Comparison of unicompartmental knee arthroplasty versus high tibial osteotomy for medial knee osteoarthritis: an updated meta-analysis of 56,000 patients. *Orthop Surg.* (2025) 17:2499–513. 10.1111/os.70049 40694375 PMC12404872

[9] Laigaard J Aljuboori SM Nikolajsen L Mathiesen O Lunn TH Lindberg-Larsen M et al. Chronic pain after primary total and medial unicompartmental knee arthroplasty for osteoarthritis: a Danish nationwide cross-sectional survey. *Acta Orthop.* (2025) 96:814–21. 10.2340/17453674.2025.44898 41189424 PMC12559960

[10] Kievit AJ Kuijer PPFM de Haan LJ Koenraadt KLM Kerkhoffs GMMJ Schafroth MU et al. Patients return to work sooner after unicompartmental knee arthroplasty than after total knee arthroplasty. *Knee Surg Sports Traumatol Arthrosc.* (2020) 28:2905–16. 10.1007/s00167-019-05667-0 31471724 PMC7471109

[11] Chakraborty A Grayson W Fricka KB Brown NM. Comparison of unicompartmental versus total knee arthroplasty in morbidly obese patients: a database analysis from 2013 to 2023. *J Arthroplasty.* (2025):S0883–5403. 10.1016/j.arth.2025.11.041 [Epub ahead of print]. 41297774

[12] Nettrour JF Ellis RT Hansen BJ Keeney JA. High failure rates for unicompartmental knee arthroplasty in morbidly obese patients: a two-year minimum follow-up study. *J Arthroplasty.* (2020) 35:989–96. 10.1016/j.arth.2019.11.003 31796233

[13] Arthur LW Jenkins C Dodd CAF Price AJ Jackson WFM Bottomley N et al. Mid-term outcomes of the fixed-bearing lateral Oxford unicompartmental knee arthroplasty. *Bone Joint J.* (2025) 107-B:432–9. 10.1302/0301-620x.107b4.bjj-2024-0977.r1 40164181

[14] Lee QJ Chang WYE Wong YC. Mid-term survivorship of fixed bearing unicondylar knee arthroplasty with no exclusion of early PFJ arthritis or ACL deficiency: analysis of clinical and radiological predictors. *J Orthop Surg (Hong Kong).* (2022) 30:10225536221141782. 10.1177/10225536221141782 36419402

[15] Burger JA Kleeblad LJ Sierevelt IN Horstmann WG Nolte PA. Bearing design influences short- to mid-term survivorship, but not functional outcomes following lateral unicompartmental knee arthroplasty: a systematic review. *Knee Surg Sports Traumatol Arthrosc.* (2019) 27:2276–88. 10.1007/s00167-019-05357-x 30689001

[16] Xu S Lim WJ Chen JY Lo NN Chia SL Tay DKJ et al. The influence of obesity on clinical outcomes of fixed-bearing unicompartmental knee arthroplasty: a ten-year follow-up study. *Bone Joint J.* (2019) 101-B:213–20. 10.1302/0301-620x.101b2.bjj-2018-0969.r2 30700121

[17] Plate JF Augart MA Seyler TM Bracey DN Hoggard A Akbar M et al. Obesity has no effect on outcomes following unicompartmental knee arthroplasty. *Knee Surg Sports Traumatol Arthrosc.* (2017) 25:645–51. 10.1007/s00167-015-3597-5 25863681

[18] Lua J Kripesh A Kunnasegaran R. Is unicompartmental knee arthroplasty truly contraindicated in an obese patient? A meta-analysis. *J Orthop Sci.* (2023) 28:1317–24. 10.1016/j.jos.2022.09.011 36336639

[19] Huang Q Zeng Y Hu Q Si H Nie Y Shen B. [Comparison of unicompartmental knee arthroplasty and total knee arthroplasty in the treatment of severe medial compartment osteoarthritis]. *Zhongguo Xiu Fu Chong Jian Wai Ke Za Zhi.* (2021) 35:1125–32. 10.1302/3114-210558 34523277 PMC8444138

[20] Liu Y Gao H Li T Zhang Z Zhang H. The effect of BMI on the mid-term clinical outcomes of mobile-bearing unicompartmental knee arthroplasty. *BMC Musculoskelet Disord.* (2022) 23:45. 10.1186/s12891-022-05001-9 35027035 PMC8756623

[21] Arirachakaran A Choowit P Putananon C Muangsiri S Kongtharvonskul J. Is unicompartmental knee arthroplasty (UKA) superior to total knee arthroplasty (TKA)? A systematic review and meta-analysis of randomized controlled trial. *Eur J Orthop Surg Traumatol.* (2015) 25:799–806. 10.1007/s00590-015-1610-9 25676725

[22] Tille E Beyer F Auerbach K Tinius M Lützner J. Better short-term function after unicompartmental compared to total knee arthroplasty. *BMC Musculoskelet Disord.* (2021) 22:326. 10.1186/s12891-021-04185-w 33810795 PMC8019176

[23] Salvi AG Valpiana P Innocenti B Ghirardelli S Bernardi M Petralia G et al. The restoration of the prearthritic joint line does not guarantee the natural knee kinematics: a gait analysis evaluation following primary total knee arthroplasty. *Arthroplast Today.* (2024) 30:101586. 10.1016/j.artd.2024.101586 39717836 PMC11665368

[24] McEwen P Omar A Hiranaka T. Unicompartmental knee arthroplasty: what is the optimal alignment correction to achieve success? The role of kinematic alignment. *J ISAKOS.* (2024) 9:100334. 10.1016/j.jisako.2024.100334 39419311

[25] Xia K Min L Xie W Yang G Yon DK Lee SW et al. Is unicompartmental knee arthroplasty a better choice than total knee arthroplasty for unicompartmental osteoarthritis? A systematic review and meta-analysis of randomized controlled trials. *Chin Med J (Engl).* (2025) 138:1568–77. 10.1097/cm9.0000000000003193 38997246 PMC12233926

[26] Shen G Shen D Fang Y Li X Cui L Wei B et al. Clinical outcomes of revision total knee arthroplasty after high tibial osteotomy and unicompartmental knee arthroplasty: a systematic review and meta-analysis. *Orthop Surg.* (2022) 14:1549–57. 10.1111/os.13311 35611758 PMC9363736

[27] Shim SJ Park YG Lee YS. Comparison of the effect of total knee arthroplasty and high tibial osteotomy on coronal pelvic and ankle-hindfoot alignment. *Knee.* (2023) 42:170–80. 10.1016/j.knee.2023.02.002 37003092

